# What's in a word: the use, misuse, and abuse of the word “persistence” in *Chlamydia* biology

**DOI:** 10.3389/fcimb.2014.00027

**Published:** 2014-03-04

**Authors:** Patrik M. Bavoil

**Affiliations:** Department of Microbial Pathogenesis, School of Dentistry, University of MarylandBaltimore, MD, USA

**Keywords:** *Chlamydia*, persistence, recurrence, commensalism, aberrant body

## Abstract

The word persistence was used by *Chlamydia* researchers almost as soon as *Chlamydia* research was born to reflect the propensity of chlamydiae to cause inapparent infection in their hosts, from birds to humans. More recently, the term persistence has been used, misused, and sometimes abused amidst *in vitro* and *in vivo* studies that aim to mimick the ability of chlamydiae to emerge from the presumed inapparent state into clinically detectable infection and disease. Here, I have attempted to provide a global perspective on the state of research on chlamydial persistence, revisiting old observations that may warrant a new look, critically evaluating more recent observations and their shortcomings, and including recent developments that may help redefine chlamydiae as pathogens—or not—of both animals and humans.

“Essentially, all models are wrong, but some are useful” (George E. P. Box).

The word persistence derives from the Latin *persistere* where *per* conveys the notion of permanence while the verb *sistere* is most frequently translated as *to stand still*. Thus, the question before us is whether the word persistence is appropriately used to describe chlamydiae that are able to “stand still permanently” in a susceptible host.

Bacterial persistence in plain English has been described often and in many different contexts. A compendium of these descriptions may be summarized as follows: Persistence is an alternative outcome of a bacterial infection whereby a subpopulation of the bacteria becomes “invisible” by variably escaping prolonged antibiotic treatment, warding off innate and adaptive immune responses, causing little or no symptoms in the infected host, and falling below the radar of the diagnostician. Cryptic, latent, covert, dormant, or silent are other terms that have been commonly used to refer to the “persistent” infection state which may consist of an altered form of the bacterium that is inherently physiologically refractory to all the above, or a normal form of the bacterium that is somehow hidden and/or effectively protected from all the above. If one takes away the notion of *infection* from these definitions, then a persistent, asymptomatic infection may closely resemble *colonization*, similar to that by a member of the microbiota, i.e., a commensal organism. Where do *Chlamydia* spp. Belong on the spectrum of commensalism and pathogenesis, and how this impacts the terminology that should be used to refer to persistence are the subject of this commentary.

From a public health point of view, the ability of *Chlamydia trachomatis* to persist is made significant by its ability to occur or recur as an acute infection since persistence on its own would be of little medical interest. In a formal sense, two basic, non-mutually exclusive scenarios should be considered: (1) a clinically detectable infection may recur from a persistent state intervening between two acute episodes; (2) alternatively, a clinically detectable infection may be induced “opportunistically” from a silent state, whereby the host has been colonized or infected at an earlier date without any symptoms or clinical signs of infection. Here, I will respectively refer to these pathways as the *persistence/recurrence* and *colonization* pathways. In either case, the previously “invisible” *C. trachomatis* population becomes “visible” to the host cell, the infected host or to the physician. However, although a distinction between these two scenarios is not usually made, they likely are biologically distinct in the manner chlamydiae become established in the host, how they exit from the “invisible” state, and also in the innate and adaptive responses they elicit, and consequent pathologies. In the absence of evidence for the colonization pathway for *C. trachomatis* to date, far more attention has been given to the persistence/recurrence mechanism owing principally to the emergence in the last two decades of several related *in vitro* models (reviewed in Wyrick, 2010) that recapitulated the basic requirements of the persistence/recurrence phenotype: the induction of infecting chlamydiae into developmental arrest upon exposure to a physiologically relevant stimulus (mimicking *in vivo* persistence), and the subsequent reversal to normal development upon removal of the stimulus (mimicking *in vivo* recurrence) in cultured cells. However, the concept of persistence did not start with human *C. trachomatis* infections; it started with the avian pathogen, *Chlamydia psittaci*.

The concept of *latent* chlamydial infection was born in the 1930s from observations by Meyer and Eddie who contrasted the frequent occurrence of the psittacosis virus, known today as *C. psittaci*, in companion birds with the relative rarity of overt disease in the birds themselves or the bird handlers (Meyer et al., 1935). Thus, the idea of persistent infection was borne out of the observation that multiple hosts may display completely different outcomes after exposure to the organism, with the majority displaying a latent infection, and a minority displaying overt disease. For infection to recur, it needs to have occurred at least once previously, and it is unclear from these studies whether the latent infected birds had a clinically detectable infection at the onset or were merely colonized. Decades after these early observations, several investigators were able to demonstrate that chlamydial development could be arrested at mid stage by either depriving the chlamydiae of essential metabolites (Bader and Morgan, 1961; Hatch, 1975) or via exposure to aminopterin (Pollard and Sharon, 1963), a folic acid competitive inhibitor and antineoplastic drug once commonly used in chemotherapy. Restitution of the required nutrients, or, in the latter case, supplementation with folinic acid, a vitamer of folate, restored the normal course of development, thereby fulfilling the essential requirements of the persistence/recurrence phenotype. These pioneering studies involved latent infection by *C. psittaci* and a link was not made then with other chlamydial diseases.

Moulder and his colleagues were the first to tackle the question of persistent/recurrent infection in a systematic experimental manner and were, indeed, first to develop *in vitro* models of persistence presumed to represent clinically observed persistence in several seminal publications. When mouse fibroblasts (L cells) were infected with high doses of *C. psittaci* 6BC, most L cells died but a small fraction survived that appeared to be persistently infected with *C. psittaci*, yet were inclusion-free by standard imaging methods of the time (Moulder et al., 1980). The cryptically infected L cells grew poorly, became resistant to super-infection (Moulder et al., 1981, 1982) and the cultures alternated between L cell expansion and chlamydial growth. A subpopulation of L cells with unique properties that enabled persistent infection by cryptic forms of *C. psittaci* was hypothesized. McCoy cell cultures persistently infected with a non-LGV strain of *C. trachomatis* were also obtained upon generating an equilibrium whereby periods of host cell propagation alternated with chlamydial growth and host cell destruction (Lee and Moulder, 1981). While Moulder and colleagues did not have the benefit of PCR, modern omics or imaging techniques, their studies described the ability of chlamydiae to maintain themselves in culture for extended periods of time, representative of a presumed “persistent state” in clinical disease, and to revert upon some unknown stimulus representative of recurrence. Unfortunately, these studies were discontinued around the eighties. Moulder did one last experiment on cryptic bodies that was a precursor of the next era of studies on chlamydial persistence/recurrence. He observed that he could delay the onset of overt chlamydial growth by shifting *C. psittaci*-persistently infected L cells to a nutrient-poor minimal medium or upon exposure to penicillin (Moulder, 1983).

A student of Moulder, Byrne, and his colleagues took the modeling of persistence/recurrence to a new level when they reproduced inducible persistence/recurrence in cultured cells using a physiologically relevant stimulus. Beatty et al. (1993) showed that in the presence of low levels of the cytokine interferon-gamma (IFN-γ), developmental growth of *C. trachomatis* serovar A stopped, producing large atypical reticulate body (RB) forms and that reversion to productive development to infectious elementary bodies (EBs) could be rescued by removal of the cytokine. IFN-γ was shown to exert its activity through tryptophan depletion upon induction of the tryptophan catabolizing enzyme indoleamine 2,3-dioxygenase (IDO) (Beatty et al., 1994), fundamentally reducing this mechanism to starvation for an essential nutrient. Similar results, including the observation of abnormally large RBs, consistent with persistence/recurrence were concurrently obtained by Pearce and his colleagues who tested the impact of starvation of several amino acids on chlamydial development (Coles et al., 1993; Pearce et al., 1994) and by Kahane and her colleagues who tested heat shock (Kahane and Friedman, 1992). Abnormally enlarged RBs similar to those observed by these groups had in fact been observed before. Over two decades earlier, Akira Matsumoto had published electron micrographs documenting the dramatic impact of penicillin on *C. psittaci* and *C. trachomatis* development (Matsumoto and Manire, 1970; Matsumoto, 1988). Similar to heat shock and tryptophan depletion, penicillin caused the formation of abnormally enlarged RBs that could be maintained in culture for as long as the host cells were maintained healthy, and that could revert to normal development to infectious EBs upon removal of the drug.

The common denominator of the early reports on chlamydial persistence/recurrence is the observed ability of a chlamydia growing in a cultured cell to withstand a variety of stresses by entering into a non-septating phase for a period of time, from which it can exit and revert to normal development upon removal of the stressor. The formation of abnormally enlarged, multinucleated (Lambden et al., 2006) aberrant RBs^1^ (aRBs) may parallel stress-induced non-dividing filamentous forms in other bacterial species that unlike *Chlamydia* are constrained by a rod-shaped peptidoglycan sacculus (e.g., *Escherichia coli, Bacillus subtilis*). Other stressors that cause multiple *Chlamydia* spp. growing *in vitro* to produce aRBs have been described since. These include chlamydiaphage superinfection of *Chlamydia caviae* (Hsia et al., 2000), co-infection with viruses (Deka et al., 2006; Borel et al., 2010) or protozoa (Romano et al., 2012, 2013), growth of *C. trachomatis* in monocytes (Koehler et al., 1997), macrophage-like (Nettelnbreker et al., 1998), or fibroblast-like synovial cells (Hanada et al., 2003), iron restriction of *C. trachomatis* (Raulston, 1997), and exposure to a variety of antibiotics (Gieffers et al., 2004). These forms have also been observed *in vivo* in varied contexts that may or may not relate to a persistent chlamydial infection (Borel et al., 2008; Pospischil et al., 2009; Rank et al., 2011; Phillips Campbell et al., 2012).

In the battleground that is an infection site, it is predictable that both the pathogen and the infected host are experiencing stress. Thus, chlamydiae undergoing a stress response should predictably appear during infection irrespective of whether the infection is acute or persistent. Indeed, aRBs can even be observed, albeit infrequently, in *in vitro* culture in the absence of any apparent stress (Tan et al., 2010). Moreover, the published literature is uneven in reporting the occurrence of typical, highly enlarged aRBs in association with stress-induced *in vitro* persistence/recurrence. In some reports, “aberrant inclusions” are referred to, confusingly implying they contain aRBs or other aberrant forms (e.g., miniature RBs), or that the inclusions themselves are aberrant (e.g., smaller vs. larger than normal). When found, the aRBs may also differ in their general appearance (e.g., Variable vacuolation). An interesting case may be that of *C. trachomatis* serovar L2 strains whereby in some stress-induced persistence systems, typical, highly enlarged aRBs are readily observed (Matsumoto, 1988; Coles et al., 1993; Harper et al., 2000; Lambden et al., 2006; Capmany and Damiani, 2010; McKuen et al., 2013) while they are not readily apparent in others (Rothermel et al., 1983; Shemer and Sarov, 1985; Huston et al., 2008; Skilton et al., 2009). While such differences may reflect experimental discrepancies or systematic differences, they globally reflect that a putative stress response-based persistence phenotype is unlikely to adhere to a single operational mode as highlighted previously by Wyrick (2010). Taken together, the molecular, cellular, and mechanistic diversity of the stress response in different *Chlamydia* strains, serovars and species growing in different cells, and the parallel diversity of conditions, which in different cells and sites of the infected host, can lead to chlamydial stress, signify that the observation of aRBs in an infected site is not sufficient to define a persistent infection. The observation that the requirements of the persistence/recurrence pathway can be fulfilled without the production of aRBs also supports that these forms are not necessary for persistence to occur.

Whether the stress response is involved in clinically observable persistence is an open question for which the model systems have not provided an answer as of yet. What is persistence? Without a clear answer to this question, it is even more difficult to answer the question of how it should be referred to. Without going into the uncertainties on persistent *C. trachomatis* genital infection in humans (i.e., recurrence from a persistent state vs. re-infection from an infected partner), an answer to this question may be provided by expanding our field of vision beyond *C. trachomatis*, to the veterinary *Chlamydia* species. All veterinary *Chlamydia* spp., including the phylogenetically close relatives of *C. trachomatis, Chlamydia muridarum*, and *Chlamydia suis* that infect mice and pigs, respectively, are first and foremost residents of the digestive tract of their host. *C. muridarum* is used experimentally to reproduce a genital infection in the mouse that replicates many features of the human *C. trachomatis* infection (Rank, 1994), but is not known to cause disease in wild mice. All veterinary *Chlamydia* spp. are transmitted primarily via the oral-fecal route and are thought to only cause disease in special circumstances, for instance if the innate or immune defenses of a given animal are weakened, or because the nearby animal population is highly infected such that individual animals are constantly re-exposed to infectious chlamydiae (e.g., abortion “storms” caused by *Chlamydia abortus* Pospischil et al., 2002). A possible explanation for the relative inattention to the possibility of an enteric phase for *C. trachomatis* may, therefore, relate to human evolution, which has witnessed a continuous reduction in the role of the fecal-oral route of transmission in the dissemination of infectious diseases in human communities. Recent studies in the mouse:*C. muridarum* model by Yeruva, Rank and colleagues have demonstrated the ability of *C. muridarum* to persist in the gastrointestinal (GI) tract of mice after oral inoculation (Yeruva et al., 2013b). These authors have further shown that the first-line antibiotic, azithromycin, while effective at clearing genital infection, was ineffective at clearing the GI infection (Yeruva et al., 2013a). Jones and colleagues first alluded to the idea that *C. trachomatis* may survive passage through the digestive tract and colonize the lower digestive tract. They observed a correlation between positive pharyngeal and rectal cultures in women who reported no history of rectal intercourse (Jones et al., 1985). These authors also observed a strong association between genital and rectal infection in these women and proposed that “autoinoculation with infected genital secretions may be the primary mechanism by which such [rectal] infections are acquired.” In contrast, Yeruva and Rank now propose that, in fact, autoinoculation may be going in the reverse direction, i.e., from the infected GI tract to the female genitalia (Rank and Yeruva, 2014). A sobering thought then is that the practice of oral sex, which is on the rise particularly in adolescent populations where chlamydial infections are also increasing, is providing the inoculum for a reservoir of *C. trachomatis* persisting in the GI tract of humans, thereby compensating for the loss of the fecal-oral route. The model proposed by Yeruva and Rank is remarkable in its simplicity and is consistent with well-known immune down-regulation mechanisms that maintain the gut microbiota and exclude pathogens. Occasional release of infectious EBs from a protected GI site and consequent autoinoculation of the genital tract would also explain the long periods of clinical “invisibility” some individuals experience.

I was asked by the editors of this special issue to attempt to provide new definitions for what *Chlamydia* researchers globally refer to as persistence. Clearly this is not possible without a better understanding of what persistence actually is. The possibility of an enteric phase for *C. trachomatis*, highlighted by the work of Yeruva and Rank, suggests that a *colonization* pathway to persistence I alluded to initially, whereby *C. trachomatis* is primarily a commensal of the GI tract that can occasionally cause disease when in the wrong place, should be seriously evaluated. Whether or not we continue to use, misuse, and abuse the word persistence in chlamydial biology may depend on it.

## Conflict of interest statement

The author declares that the research was conducted in the absence of any commercial or financial relationships that could be construed as a potential conflict of interest.

## Footnotes

^1^Herein referred to as aberrant RBs or aRBs; although these have been variably named aberrant bodies (ABs), or persistent bodies (PBs), this should be discouraged as it convey that these forms have uniform properties across multiple stressors.
